# Lung sonography

**DOI:** 10.1186/1824-7288-40-S1-A7

**Published:** 2014-08-11

**Authors:** Paolo Tomà

**Affiliations:** 1 Department of Imaging, Bambino Gesù Children’s Hospital, IRCCS, Piazza S. Onofrio, 00165 Rome, Italy

## 

New extensive use of thoracic ultrasound (TUS) takes information also from physical acoustic phenomena that are not directly convertible into images of the human body[1].This tendency also takes into account the classic patterns (based on the presence of an adequate acoustic window) emphasizing the role of TUS as an all-in-one approach in many conditions. We stated our perplexities [2] and we maintain that great caution is warranted when this procedure is used. The evidences on neonatology and paediatrics are based on few articles with different biases i.e.:

No evaluation on technical issues feasibly (the generation of artefacts is conditioned by the time gain compensation setting, the ratio of probe curvature to the curvature of the lung surface, and whether or not movement artefact suppression and tissue harmonics are used), no comparison of the results of TUS and those of a reference standard (i.e. CXR);

Diagnostic access bias (few cases, small hospital), bias from reader/training experience on CXR (no paediatric radiologists);

Imaging analysis bias i.e. absence of preliminary definition of the methods for imaging (CXR) interpretation, selection bias (patients from birth to adult undergoing chest radiography for suspected community acquired pneumonia).
